# Hydroxyapatite/MCM-41 and SBA-15 Nano-Composites: Preparation, Characterization and Applications

**DOI:** 10.3390/ma2041508

**Published:** 2009-09-30

**Authors:** Oscar A. Anunziata, Maria L. Martínez, Andrea R. Beltramone

**Affiliations:** Grupo Fisicoquímica de Nuevos Materiales, Centro de Investigación y Tecnología Química (CITeQ), Facultad Córdoba, Universidad Tecnológica Nacional, 5016 Córdoba, Argentina; E-Mails: mmartinez@scdt.frc.utn.edu.ar (M.L.M.); abeltramone@scdt.frc.utn.edu.ar (A.R.B.)

**Keywords:** HaP**/** MCM-41, HaP/ SBA-15, nanocomposites, F^-^ retention, water

## Abstract

Composites of hydroxyapatite (HaP) and highly ordered large pore mesoporous silica molecular sieves such as, Al-SBA-15 and Al-MCM-41 (denoted as SBA-15 and MCM-41, respectively) were developed, characterized by XRD, BET, FTIR, HRTEM and NMR-MAS, and applied to fluoride retention from contaminated water. The proposed procedure by a new route to prepare the HaP/SBA-15 and HaP/MCM-41, composites generates materials with aluminum only in tetrahedral coordination, according to the ^27^Al NMR-MAS results. Free OH^-^ groups of HaP nanocrystals, within the hosts, allowed high capacity fluoride retention. The activity of fluoride retention using HaP/MCM-41 or HaP/SBA-15 was 1-2 orders of magnitude greater, respectively, than with pure HaP.

## 1. Introduction

Calcium phosphate apatites are compounds of the formula Ca_5_(PO_4_)_3_X, where X can be a F^−^ (fluorapatite, FaP), OH^−^ (hydroxyapatite, HaP) or a Cl^−^ ion (chlorapatite). One ion is replaced by another of the same sign but of different charge. Neutrality is maintained by substitutions of ions with dissimilar charges or vacancies [1].

Fluoridated calcium hydroxyapatites have been studied in relation to their physico-chemical properties [2,3,4,5,6]. It is well known that fluoride is one of the elements contained in biological apatites as trace amounts, which strongly modifies their crystallinity and their solubility. Porous hydroxyapatite biomaterials have a great stability and a good biocompatibility. They can be used as composite biomaterials for their ability to form a strong chemical bond with natural bones.

Laghzizil *et al*. [5,6] enhanced the fluoride adsorption capacity onto hydroxyapatite (HaP) prepared in a highly porous form using a modified chemical wet method. Besides, they have also analyzed the effect of the F^-^ ions on the crystallinity and electrical properties of hydroxyapatite biomaterials. Moreover, Dalas *et al.* [7] have studied the crystallization of hydroxyapatite on polymers, containing ‑C-N groups, from supersaturated solutions of HaP. Consequently, this method was particularly useful to study the formation of new phases on the substrates in which HaP was deposited, for example the growth of hydroxyapatite on silica gels in the presence of organic additives [8]. In other research, nanosized hydroxyapatite particles have been successfully synthesized from microemulsions stabilized by a biodegradable surfactant [9,10]. These particles possess powder characteristics that make them superior in many composites applications. The microemulsion-derived hydroxyapatite powders exhibit a high specific surface area, lowered degree of particle agglomeration and narrow particle size distribution [10].

On the other hand, the synthesis of mesoporous hydroxyapatite was reported by several authors [11,12,13,14,15,16,17], e.g., Tang *et al*. [18] described a simple and new method for the preparation of hydroxyapatite porous biomaterials with a uniform pore size distribution by sintering the mixture of HaP powders and monodispersed polystyrene microspheres.

In a previous work, we published our first report on the activity of HaP/ MCM-41 and HaP-BEA composites for fluoride retention [19]. We developed a technique of preparation of nanocrystalline HaP (*ex-situ*) and in the presence of the respective hosts, forming *in situ* composites. We also compared the capacity of F^−^ retention from contaminated water, with respect to a commercial sample.

In the present work, we prepare composites of hydroxyapatite (HaP) and highly ordered large pore mesoporous silica molecular sieve such as Al-SBA-15 and Al-MCM-41. We correlate fluoride retention, from contaminated water, with the physicochemical properties of HaP/MCM-41 and HaP/SBA-15 nanocomposites. Our first results concerning the development of SBA-3, SBA-15 and SBA-1 was recently reported [20].

## 2. Results and Discussion

### 2.1. XRD and BET studies

The surface area of the hydroxyapatite commercial sample (CHaP), measured by the single-point BET (N_2_) method, was 69 m^2^/g. The surface areas were 1,140 m^2^/g for MCM-41, 960 m^2^/g for HaP/ MCM-41; 1,250 and 987 m^2^/g for SBA-15a and Hap/SBA-15a; 1,200 and 960 m^2^/g for SBA-15b and Hap/SBA-15b, respectively, and 85 m^2^/g for HaP synthesized by us. The pore diameters of the hosts were: 5.8, 9.6 and 9.8 nm for MCM-41, SBA-15a and SBA-15b, respectively. The composite HaP/MCM-41 and HaP/SBA-15 isotherms show a residual pore volume of 0.45 mL per gram of MCM-41 host, and 0.80-0.85 mL per gram for SBA-15a-b (see Table 1).

**Table 1:** Textural and structural properties of the calcined hosts and composites.

Sample	Si/Al^a^	a_o_* (nm)	Area m^2^/g	Pore Vol. mL/g	Diameter ** pore (nm)	Wall thickness*** (nm)
MCM-41	25	2.4	1140	0.80	5.80	1.1
SBA-15a	50	11.3	1250	1.32	9.60	2.2
SBA-15b	32	11.7	1200	1.26	9.80	2.4
HaP/MCM-41	25	2.4	960	0.45	3.25	1.1
HaP/SBA-15a	50	11.3	987	0.80	7.50	2.2
HaP/SBA-15b	32	11.7	960	0.85	7.20	2.4

^a^ By ICP. (*) hexagonal: a_o_ = 2 d_100_/√3; (**) D ≅ 4V/A; (***) E = a_o_ - D, (according to Ref. [21]).

**Figure 1:**
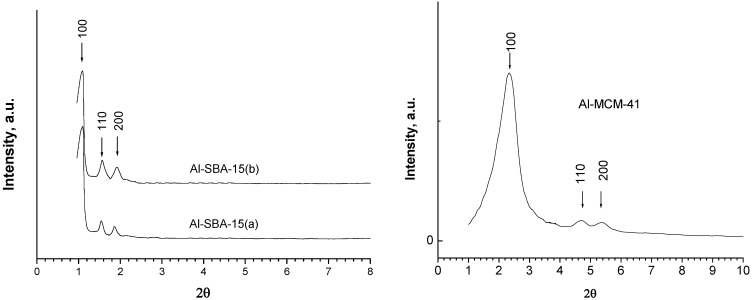
XRD of Al-SBA-15 and Al-MCM-41.

The XRD for Na-MCM-41, indicates a signal (hkl: 100) corresponding to a hexagonal structure of the mesoporous materials, at 2θ = 1.99-2.08° and ao = 4.9-5.1 nm. The low intense signals at long-range order, 110 and 200, at 2θ = 4.66° and 5.30°, respectively, are characteristics of highly ordered hexagonal structure (see Figure 1). In the case of SBA-15, the main signal appears at 1.2°(2θ) and shifts to lower angles (0.9° (2θ)) with the incorporation of Al in the case of Al-SBA-15 (see Figure 1), in agreement with literature [22,23].

**Figure 2:**
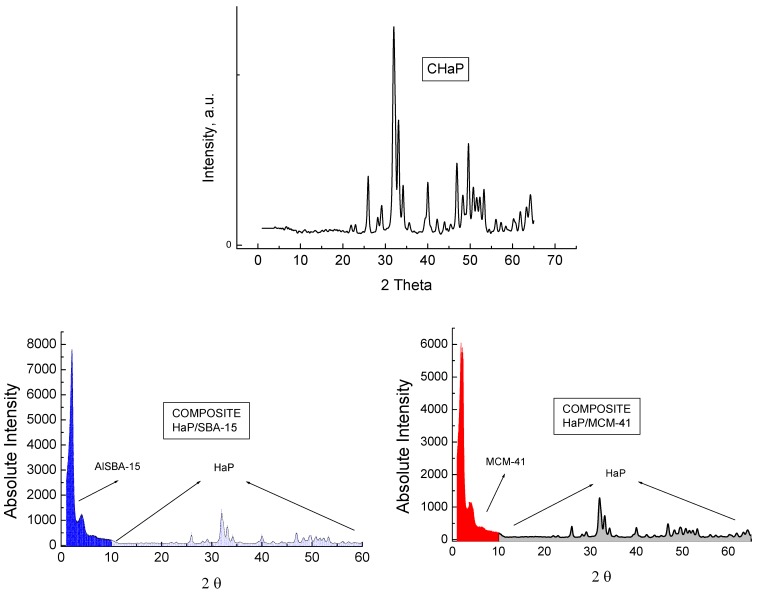
XRD of CHaP, HaP/SBA-15 and HaP/MCM-41.

The pattern diffraction peaks confirm a high crystallinity or long-range order structure in all nanostructured hosts. The XRD pattern of pure HaP prepared *ex-situ* by us, HaP/MCM-41 and HaP/SBA-15 composites are illustrated in Figure 2.

### 2.2. FTIR studies

**Figure 3:**
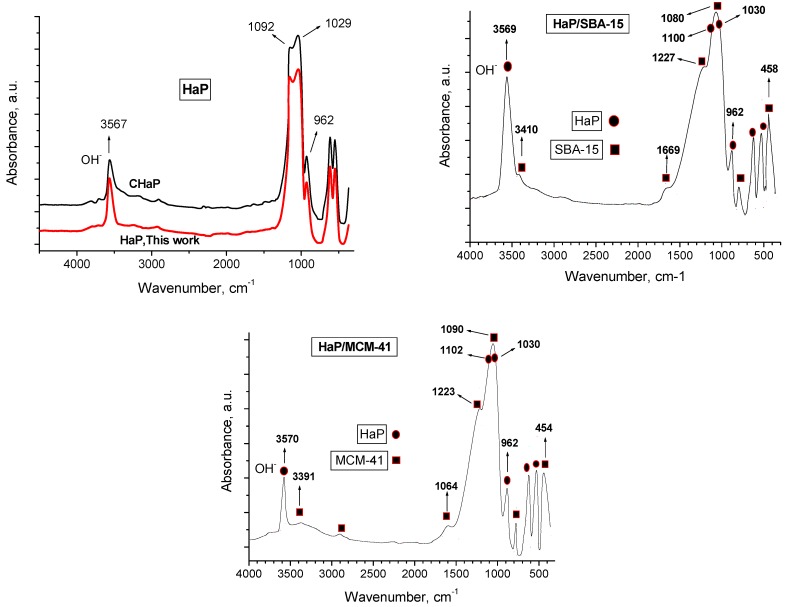
FTIR of hydroxyapatite commercial sample (CHaP), nanosized HaP prepared in this work, HaP/SBA-15b and HaP/MCM-41 nanocomposites.

FTIR data of a pure commercial hydroxyapatite sample (CHaP), HaP/MCM-41 and HaP/SBA-15 with the assigned bands (prior to the retention of F^-^) are shown in Figure 3. In ther HaP spectrum, the P–O stretching IR mode appears at ~ 962 cm^−1^ and the PO_4_ region appears as a very strong bands at ~1,029 cm^−1^ and at ~1,092 cm^−1^, whereas the band at 3,567 cm^−1^ is assigned to OH stretching mode [1]. The well defined bands at 650, 610 and 564 cm^−1^ are attributed to the components of asymmetrical deformation O-P-O. The identification of the bands was difficult in the case of HaP/MCM-41 and HaP/SBA-15 composites, with HaP crystals (in the nm range). FTIR of HaP/SBA-15 (Figure 3) shows bands corresponding to SBA-15, at 1,080 and 1,227 cm^−1^ (T–O asymmetric stretching, internal and external respectively), the band at 800 cm^-1^ (T–O symmetric stretching) are due to TO_4_ vibrations (T = Si). Some authors [24,25] have assigned this band to the Al–O–Si bending, indicating the incorporation of Al into SBA-15 in final samples. Such positive shifts in frequencies (from 798 cm^-1^ in the as-synthesized sample), would reflect the formation of new Si–O–Si and Si–O–Al bridges during calcinations. In this way, it is probably due to an increased network cross-linking [26], and would account for the lattice contraction and structural stabilization that Al-MCM-41 and Al-SBA-15 undergoes upon template removal and calcination process. The signal at 458 cm^−1^ is assigned to a bending of T-O. In the same way, FTIR of HaP/MCM-41 shows bands corresponding to MCM-41, at 1,090, 1,223 cm^−1^ (T–O asymmetric stretching, internal and external respectively) and 800 cm^−1^ (T–O symmetric stretching) are due to TO_4_ vibrations (T = Si), assigned to the bending Si–O–Si and a band at 454 cm^−1^ due to the bending of T-O. The band at 1,630 cm^−1^ ascribed to the Si–O stretching overtone also appears clean with evacuation of the hosts at 400 °C. The behavior is similar for both samples. By FTIR of the composite in the OH stretching zone, a strong signal at 3,567 cm^−1^ due to OH^-^ of HaP is observed [27]. The integrated absorbance of this band per mg of HaP (see Section 3.2), for each sample is CHaP: 0.125; nanosized HaP prepared in this work: 0.31; HaP/MCM-41(30 wt% of HaP): 0.51 and HaP/SBA-15b (35 wt% of HaP): 0.69. This band must remain intact (without any interaction with the hosts), in order that the capacity of F^-^ retention of composite do not be altered as long as possible (Figure 3).

### 2.3. NMR-MAS studies

**Figure 4:**
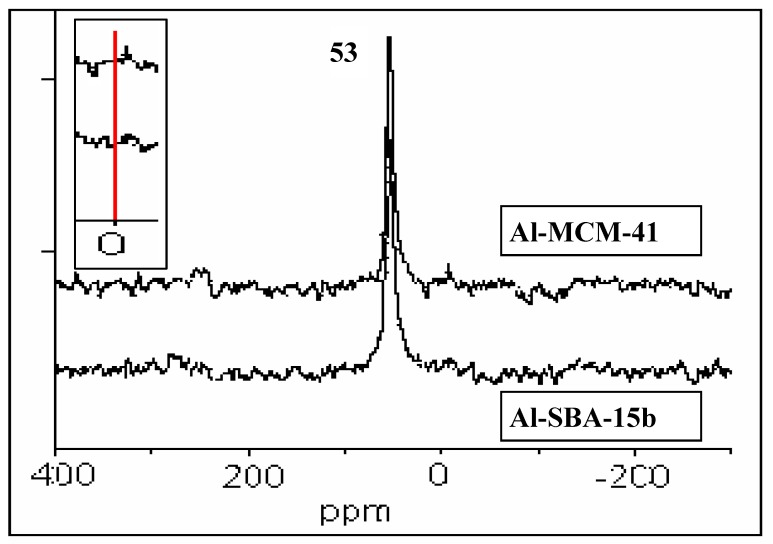
^27^Al MAS-NMR spectra of Al-MCM-41 and Al-SBA-15b.

^27^Al-NMR-MAS results of the samples [20,28], showed a intense peak at 53 ppm, assigned to Al^IV^_Td_ form, a very low signal at 0 ppm due to octahedral extra framework aluminum (Al^VI^_Oc_), can be seen in Figure 4 (see inset spectrum) for Al-SBA-15b and Al-MCM-41 materials.

### 2.4. HRTEM and SEM studies

**Figure 5:**
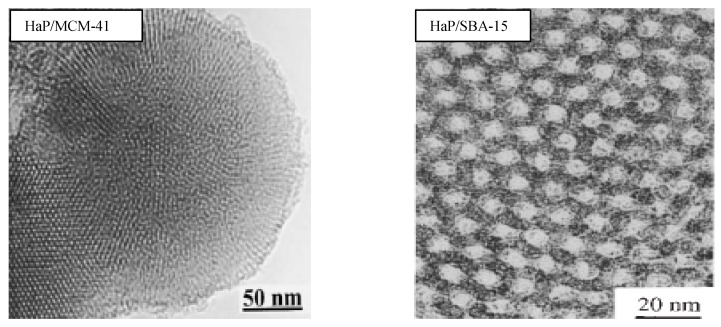
HRTEM of HaP/MCM-41 and HaP/SBA-15b.

The HRTEM images illustrated in Figure 5, reveal the existence of a long-range hexagonal arrangement of nanosized mesopores. The higher order reflections are still discernable clearly in the sample HaP/MCM-41 and HaP/SBA-15 compared with the HRTEM of the hosts reported in literature [19,29]. Thus, the nanosized crystals of HaP are within the mesostructure of the hosts.

**Figure 6:**
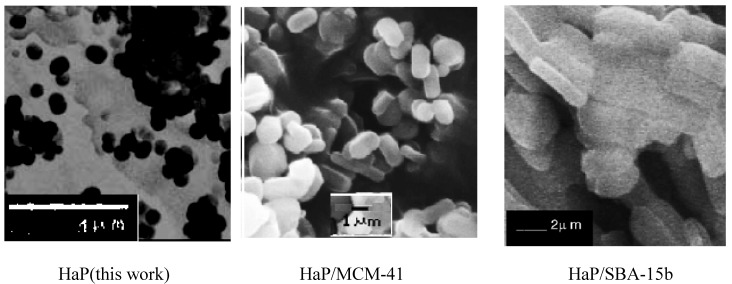
SEM of HaP, HaP/MCM-41 and HaP/SBA-15b.

The size and shape of the samples indicate good morphology of the crystals. HaP-SBA-15 images reveal that it consists of many rope-like domains with relatively uniform sizes of 1.5–2 μm, without other phases (clusters of HaP crystals) as well as in HaP-MCM-41 microphotographs, but with micellar rod-like shape hexagonal crystals, with size of 1.5 × 2 .2 µm (see Figure 6), in agreement with HRTEM data showed in Figure 5.

### 2.5. Fluoride retention

**Figure 7:**
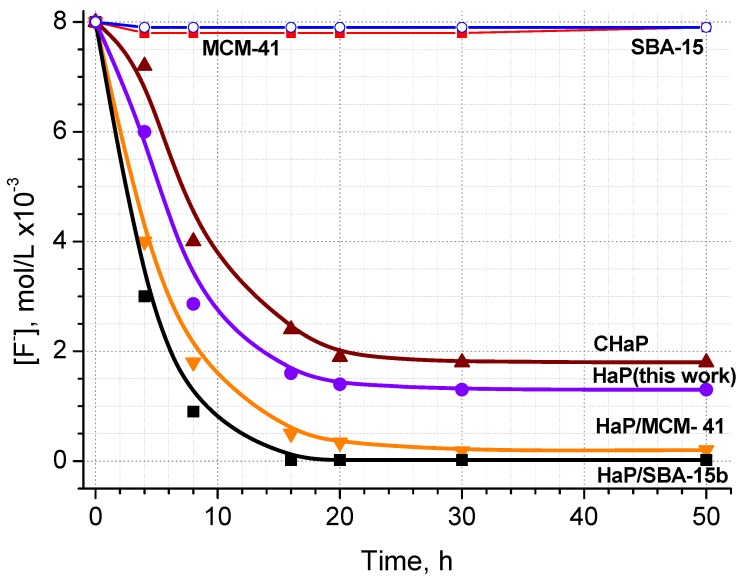
F^-^ retention capacity from contaminated water of the samples vs. time.

Figure 7 shows the F^-^ retention capacity of the samples. The method used for the host inclusion (not found in literature) seems to be adequate, since the OH^-^ groups of HaP were not blocked. In the case of HaP (*ex-situ*), its lower crystal size has favored the F^-^ retention, compared with the commercial sample. MCM-41 and SBA-15b act as supports to anchor the HaP crystals, on a nanometer scale (<3 nm and 10 nm, respectively), with higher fluoride retention from contaminated water, in correspondece with the data showed in FTIR studies (see Figure 3). In Figure 7, we can see that the fluoride retention by the hosts is not significant. The results demonstrated first, a fast retention of fluoride from 0 to 10 hours and then decaying to the stationary state, in about 25 hours. The final concentration of fluoride ion was 0.15 and 0.02 × 10^−3^ M, for HaP/MCM-41 and HaP/SBA-15 respectively. In this way, Table 2 shows the diminution of the OH^-^ band of HaP (signal at 3567 cm^-1^ for CHaP and nanosized HaP prepared in this work and 3,569-3,570 cm^-1^ for HaP/SBA-15 and HaP/MCM-41 nanocomposites), as a function of time on stream, for the data shown in Figure 7.

The results are shown as percentage of the OH^-^ band, in absorbance units for each sample, which remains unalterable during the fluorides retention test, considering the 100% of the absorbance of this signal before the beginning of the test.

As can be seen, the nanocomposites of HaP/MCM-41 and HaP-SBA-15, retain fluoride with better performance, even after 20 h of evaluation, than the HaP crystals. The best performance of HaP/SBA-15 with respect to HaP/MCM-41, could be due to a high dispersion with lower size of HaP nanocrystals (linked to its higher surface area and pore volume). Taking account that SBA-15 material has higher amount of silanol groups than MCM-41 [20], the condensation of adjacent silanol groups (Si–(OSi)_3_–OH) forms siloxane species, which might anchor Ca^2+^, in order to make available sites for the HaP nanocrystals nucleation. Thus, as the silanol sites increase, the possibility to generate more sites for the growth of HaP crystals increases, these results are in agreement with Díaz *et al.* [30].

**Table 2:** OH^-^ bands of HaP, in % Absorbance Units, at different times after fluoride retention.

Samples	OH^-^ bands at 3667-3670 cm^-1^, Absorbance Units, %*			
	Time, h			
	4	10	20	50
CHaP^a^	80	18	6	2
HaP^b^	75	23	8	3
HaP/MCM-41	73	57	40	41
HaP/SBA-15b	71	60	45	43

* See the text; ^a^ Commercial HaP; ^b^ HaP this work.

## 3. Experimental Section

### 3.1. Host synthesis

Al-SBA-15 was synthesized using 15-crown-15, (PEO15, Aldrich) as a co-polymer mono block and cetylpyridinium bromide (BDH 95%) as surfactant, TEOS (Aldrich 99%) and NaAlO_2_ (Aldrich 99%)_,_ as silica and aluminum source, respectively; as described elsewhere [20]. The final Si/Al ratios determined by ICP of the samples were 50-33 (denoted as SBA-15a and SBA-15b). Al-MCM-41 was developed by a new technique [28]. An aqueous solution of NaAlO_2_ was added to the mixture of silica source (Ludox TM-40 colloidal silica, Aldrich, 40% suspension in water) and aqueous TMAOH (tetramethylammonium hydroxyde). Then, both aqueous solutions of CTABr (cetyltetra-methylammonium bromide) and NaH_2_PO_4_ were added to the synthesis, then mixtured and stirred for 30 min at 20 ºC. The final gel mixtures were refluxed under stirring for a period of 24 h. The Si/Al molar ratio was 30, determined by ICP for the final catalytic material, denoted as MCM-41.

### 3.2. Preparation of the composites

HaP *ex-situ*, was prepared using CaCl_2_•2H_2_O (a) and K_2_HPO_4_ (b), and doubly distilled water. Solutions of variable concentrations were used: 1-0.51 M of CaCl_2_ and 1.8-2.3 M of K_2_HPO_4_, at pH 8-9. Solution (b) is added to solution (a) in a stirred Pyrex vessel at 37 ºC, and left for 6 h, obtaining a calcium/phosphate molar ratio of 1.7 in order to have the stoichiometric ratio of HaP, with ionic strength, I = 0.16 mol·L^-1^. The pH was adjusted to the required value by the slow addition of KOH solution. During the reaction, CO_2_ was excluded by bubbling with presaturated N_2_ gas. To prepare HaP/host (HaP-*in-situ*), the same procedure was followed, SBA-15 and MCM-41 were added at the first 0.5 h of the total reaction time of the preparation. The suspensions were vigorously stirred for 4 h, at 60 ºC, filtered, washed with triple-distilled carbon dioxide-free water, and then dried at 100 ºC for 4 h. The HaP *ex-situ* and composites were activated by heating at 500 ºC in N_2_ flow for 10 h, then calcined up to 500 ºC at a heating rate of 2 ºC/min from 100 ºC for 2h. Commercial hydroxyapatite (CHaP) also was used in this study, provided by Bio-gel HTP, marketed by BIO-RAD®. The HaP content in the composite was determined by ICP following the ratio of Ca/P and Ca/Si. From Ca/P we determined the stoichiometric ratio of HaP and with Ca/Si, the amount of HaP in the composites. Thus, HaP content for HaP/MCM-41 and HaP/SBA-15 composites were 30 and 35 wt%, respectively.

### 3.3. Fluoride retention essay

Solutions of contaminated water with fluoride were prepared, using a Teflon device with magnetic stirring, specially designed to bubble N_2_ in order to avoid CO_2_ contamination at 25 ºC. The pH of the solutions was measured with a Mettler pH meter with combination glass electrodes; the instrument was calibrated with buffers of pH = 4 and 7.5. Ion F^-^ concentration was determined using a specific electrode for F^-^, dynamic range between 1 to 300 ppm. In addition, F^-^ traces were followed by FTIR. Experimental conditions: 2.3 g of HaP in 100 mL of NaF solution with initial concentration of 8 × 10^-3^ M. The weight of the materials employed was normalized on HaP base.

### 3.4. Characterization

Nitrogen adsorption of the samples was measured with and ASAP 2010 Micromeritics apparatus. Elemental analysis was performed by inductively coupled plasma-atomic emission spectroscopy (VISTA-MPX) operated with high frequency emission power of 1.5 kW and plasma airflow of 12.0 L/min. The diffraction patterns were performed with with a Philips X’Pert PRO PANalytical diffractometer under Cu Kα radiation (λ = 1.5418). The diffraction data was collected by using a continuous scan mode with a scan speed of 0.02° (2 θ)/min. FTIR spectra of the samples were obtained using wafers of HaP and the composites in KBr employing a vacuum cell with special KBr windows and a JASCO 5300 Fourier Transform Spectrometer. Prior to the FTIR experiments, the samples were degassed (p < 10^-3^ Pa) at 400 ºC for 4 h. ^27^Al MAS-NMR spectra were taken on a BRUKER MSL300 spectrometer operating at 78.2 MHz for ^27^Al. We used a BRUKER MAS 300WB CP1H-BBWH. VTN-BL4 probe with 4 mm o.d. zirconia rotors. The size and shape of the crystals were determined by SEM in a PHILIPS-SEM 501B. High-resolution transmission electron microscopy (HRTEM) images of a few representative samples were collected using a JEOL-200 CX electron microscope. Composites samples were mounted on a microgrid carbon polymer, supported on a copper grid, by placing a few droplets of a suspension of the sample in water followed by drying at ambient conditions.

## 4. Conclusions

SBA-15 and MCM-41 were successfully developed. The materials have good structural and textural properties. They are useful as hosts incorporating nanocrystals of hydroxyapatite, forming active composites of HaP/MCM-41 and HaP/SBA-15. According HRTEM studies, HaP nanocrystals are within the hosts, and not on the external surface, indicating good incorporation of nano-crystals in the host, with sizes of pores higher than 4 nm. Fluoride retention is a function of surface area and pore diameter of the hosts (SBA-15 and MCM-41), that allow the anchoring of the HaP nanocrystals, leaving OH^-^ groups free. The capacity for fluoride retention of the HaP/hosts increases one and two order of magnitude with respect to pure HaP. Thus, we have developed useful nanocomposites of HaP/mesostructured materials, which allow efficient retention of fluoride from contaminated water.
